# Senolytic drugs, dasatinib and quercetin, attenuate adipose tissue inflammation, and ameliorate metabolic function in old age

**DOI:** 10.1111/acel.13767

**Published:** 2023-01-13

**Authors:** Md Torikul Islam, Eric Tuday, Shanena Allen, John Kim, Daniel W. Trott, William L. Holland, Anthony J. Donato, Lisa A. Lesniewski

**Affiliations:** ^1^ Department of Nutrition and Integrative Physiology The University of Utah Salt Lake City Utah USA; ^2^ Geriatric Research Education and Clinical Center Veteran's Affairs Medical Center‐Salt Lake City Salt Lake City Utah USA; ^3^ Division of Cardiology, Department of Internal Medicine The University of Utah School of Medicine Salt Lake City Utah USA; ^4^ Department of Kinesiology The University of Texas at Arlington Arlington Texas USA; ^5^ Division of Geriatrics, Department of Internal Medicine The University of Utah School of Medicine Salt Lake City Utah USA; ^6^ Department of Biochemistry The University of Utah Salt Lake City Utah USA; ^7^ Nora Eccles Harrison Cardiovascular Research and Training Institute The University of Utah Salt Lake City Utah USA

**Keywords:** aging, dasatinib, inflammation, metabolic function, quercetin, senescence, senolytics, immune cells

## Abstract

Aging results in an elevated burden of senescent cells, senescence‐associated secretory phenotype (SASP), and tissue infiltration of immune cells contributing to chronic low‐grade inflammation and a host of age‐related diseases. Recent evidence suggests that the clearance of senescent cells alleviates chronic inflammation and its associated dysfunction and diseases. However, the effect of this intervention on metabolic function in old age remains poorly understood. Here, we demonstrate that dasatinib and quercetin (D&Q) have senolytic effects, reducing age‐related increase in senescence‐associated β‐galactosidase, expression of *p16* and *p21* gene and P16 protein in perigonadal white adipose tissue (pgWAT; all *p* ≤ 0.04). This treatment also suppressed age‐related increase in the expression of a subset of pro‐inflammatory SASP genes (*mcp1, tnf‐α, il‐1α, il‐1β, il‐6, cxcl2,* and *cxcl10*), crown‐like structures, abundance of T cells and macrophages in pgWAT (all *p* ≤ 0.04). In the liver and skeletal muscle, we did not find a robust effect of D&Q on senescence and inflammatory SASP markers. Although we did not observe an age‐related difference in glucose tolerance, D&Q treatment improved fasting blood glucose (*p* = 0.001) and glucose tolerance (*p* = 0.007) in old mice that was concomitant with lower hepatic gluconeogenesis. Additionally, D&Q improved insulin‐stimulated suppression of plasma NEFAs (*p* = 0.01), reduced fed and fasted plasma triglycerides (both *p* ≤ 0.04), and improved systemic lipid tolerance (*p* = 0.006). Collectively, results from this study suggest that D&Q attenuates adipose tissue inflammation and improves systemic metabolic function in old age. These findings have implications for the development of therapeutic agents to combat metabolic dysfunction and diseases in old age.

## Graphical abstract

In this study, we show that the administration of a senolytic drug cocktail of dasatinib and quercetin reverses age‐related increases in senescence and inflammation in the adipose tissue. This drug treatment also improves metabolic function in old age. Results from this study have implications for developing new drugs to treat metabolic dysfunction and associated diseases such as diabetes.

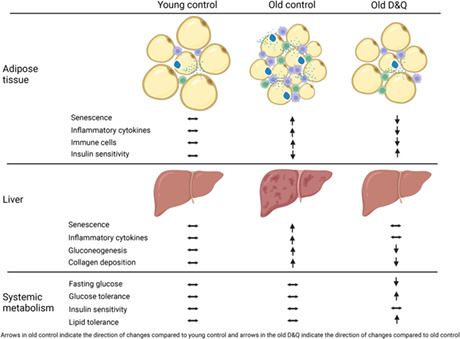

## 1 INTRODUCTION

Advancing age is accompanied by metabolic dysfunction such as an impairment in glucose and lipid metabolism (Chia et al., 2018; Petersen et al., 2017; Rines et al., 2016). The mechanisms that underlie metabolic dysfunction in old age are multifaceted (Islam et al., 2020). However, emerging evidence suggests that metabolic dysfunction in old age is concomitant with the accumulation of senescent cells across many organs and tissues including adipose tissue and liver (Borghesan et al., 2020; Xu, Palmer, et al., 2015; Yousefzadeh et al., 2020). Cellular senescence is a consequence of a multitude of stresses such as DNA damage, detrimental mutations, telomere uncapping, metabolic dysfunction, and inflammation (Bloom et al., 2022; Borghesan et al., 2020; Gorgoulis et al., 2019; Herranz & Gil, 2018). These stressors activate p53/p21 and/or Rb/p16 tumor suppressor pathways resulting in marked alterations in gene expression and cell signaling that lead to permanent arrest of the cell cycle, termed senescence (Bloom et al., 2022; Borghesan et al., 2020; Herranz & Gil, 2018). Senescent cells accumulate in multiple tissues and organs with advancing age and act as a mediator of inflammation (Borghesan et al., 2020; Gorgoulis et al., 2019). For example, senescent cells secrete a host of inflammatory cytokines, chemokines, proteases, and tissue factors, that are collectively known as the senescence‐associated secretory phenotype (SASP; Bloom et al., 2022; Borghesan et al., 2020; Gorgoulis et al., 2019). Senescent cells and the SASP result in a chronic sterile inflammation with advancing age and contribute to numerous age‐related pathologies (Borghesan et al., 2020; Gorgoulis et al., 2019).

Senescent cells and immune cells demonstrate feedback interactions. For example, senescent cells play a critical role in neonatal development, wound healing, and organ repair by activating a host of signaling cascades (Wilkinson & Hardman, 2020). After senescent cells execute these functions, immune cells such as macrophages and T cells recognize them via cell surface receptors and clear them from tissues, preventing excessive accumulation (Kale et al., 2020; Prata et al., 2018). While an interaction between senescent cells and immune cells is necessary for many physiological processes, an impairment in this interaction may amplify inflammation leading to detrimental consequences (Prata et al., 2018). Adipose tissue from both obese and old mice demonstrates elevated senescence burden and immune cell infiltration that is linked to impairments in glucose metabolism (Borghesan et al., 2020; Kalathookunnel Antony et al., 2018). Interestingly, elimination of senescent cells concomitantly reduces adipose tissue macrophage in both human and mice in the setting of obesity (Hickson et al., 2019; Palmer et al., 2019). Likewise, it has been demonstrated that the age‐related impairments in glucose metabolism are associated with adipose tissue T‐cell infiltration (Bapat et al., 2015; Khan et al., 2020; Stout et al., 2017). Moreover, interventions attenuating adipose tissue T‐cell infiltration improve metabolic function in old mice, further supporting a critical role of adipose tissue immune cells in metabolic function in old age (Bapat et al., 2015; Feuerer et al., 2009; Trott et al., 2021). However, the interactions between senescent cells and immune cells, and particularly, if clearing senescent cells can attenuate the abundance of immune cells in the adipose tissue remains unknown.

In recent years, targeting senescent cells to alleviate age‐related diseases has become a popular and rapidly growing field (Borghesan et al., 2020; Gorgoulis et al., 2019). Numerous studies have demonstrated that pharmacological agents, known as senolytics and senomorphics that respectively eliminate senescent cells and antagonize SASP, improve many age‐related diseases (Borghesan et al., 2020; Xu et al., 2018; Zhu et al., 2015). Likewise, evidence exists that genetic clearance of p16‐positive senescent cells prevents age‐related lipodystrophy (Xu, Palmer, et al., 2015). Additionally, a senomorphic agent, ruxolitinib, that inhibits senescent adipocyte‐secreted activin A, improved glucose tolerance in old mice (Xu, Palmer, et al., 2015). Collectively, these findings suggest that senescent cells can be targeted to ameliorate metabolic dysfunction in old age. Dasatinib and quercetin (D&Q) are two of the most studied senolytic drugs and have demonstrated promise in improving age‐related pathophysiological dysfunctions in human and mice (Hickson et al., 2019; Wissler Gerdes et al., 2020; Xu et al., 2018). Dasatinib is a tyrosine kinase inhibitor that has previously been approved by the FDA for treating myeloid leukemia (Kirkland & Tchkonia, 2020; Wissler Gerdes et al., 2020). Quercetin is a naturally occurring flavonoid compound that induces apoptosis in senescent endothelial cells (Kirkland & Tchkonia, 2020; Wissler Gerdes et al., 2020). Together, D&Q has been demonstrated to be effective in clearing senescent cells by inducing apoptosis in multiple tissues and are currently being investigated for treating human diseases in several clinical trials (Kirkland & Tchkonia, 2020). Recent preclinical studies have revealed that D&Q improves obesity‐induced glucose intolerance and insulin resistance in mice (Palmer et al., 2019; Sierra‐Ramirez et al., 2020), suggesting that D&Q may serve as a clinically relevant therapeutic regimen to combat metabolic dysfunction. However, the effects of D&Q on metabolic function in old age remain unexplored.

In this study, we tested the hypotheses that intermittent administration of D&Q will (1) attenuate markers of senescence in metabolic tissues, (2) diminish adipose tissue inflammatory SASP and abundance of T cells as well as macrophages, and (3) improve metabolic function in old age. To test these hypotheses, we treated old mice with D&Q and examined expression of senescence markers and a subset of inflammatory SASP factors in major metabolic tissues such as perigonadal adipose tissue (pgWAT), liver, and skeletal muscle. We also examined T lymphocyte and macrophages in the pgWAT and comprehensively assessed systemic metabolic function using glucose‐, insulin‐, pyruvate‐, and intralipid‐tolerance tests, glucose‐stimulated insulin secretion assays, insulin‐stimulated suppression of plasma‐free fatty acids and plasma triglycerides measurements. Additionally, we assessed protein and gene expression of key enzymes and proteins of the gluconeogenic and insulin signaling pathways.

## 2 METHODS

### 2.1 Animals

Male C57BL/6 mice were obtained from the National Institute on Aging colony maintained at Charles River Inc. Young mice were purchased from Charles River Inc. All mice were maintained at the Salt Lake City VA Medical Center's Animal Facility in the standard shoebox cages on a 12:12 light: dark cycle with water and food ad libitum. All animal procedures conform to the Guide for the Care and Use of Laboratory Animals (Carbone, 2012) and were approved by the University of Utah and Salt Lake City VA Medical Center Animal Care and Use Committee.

### 2.2 Senolytic drugs and vehicle treatments

**FIGURE 1:**
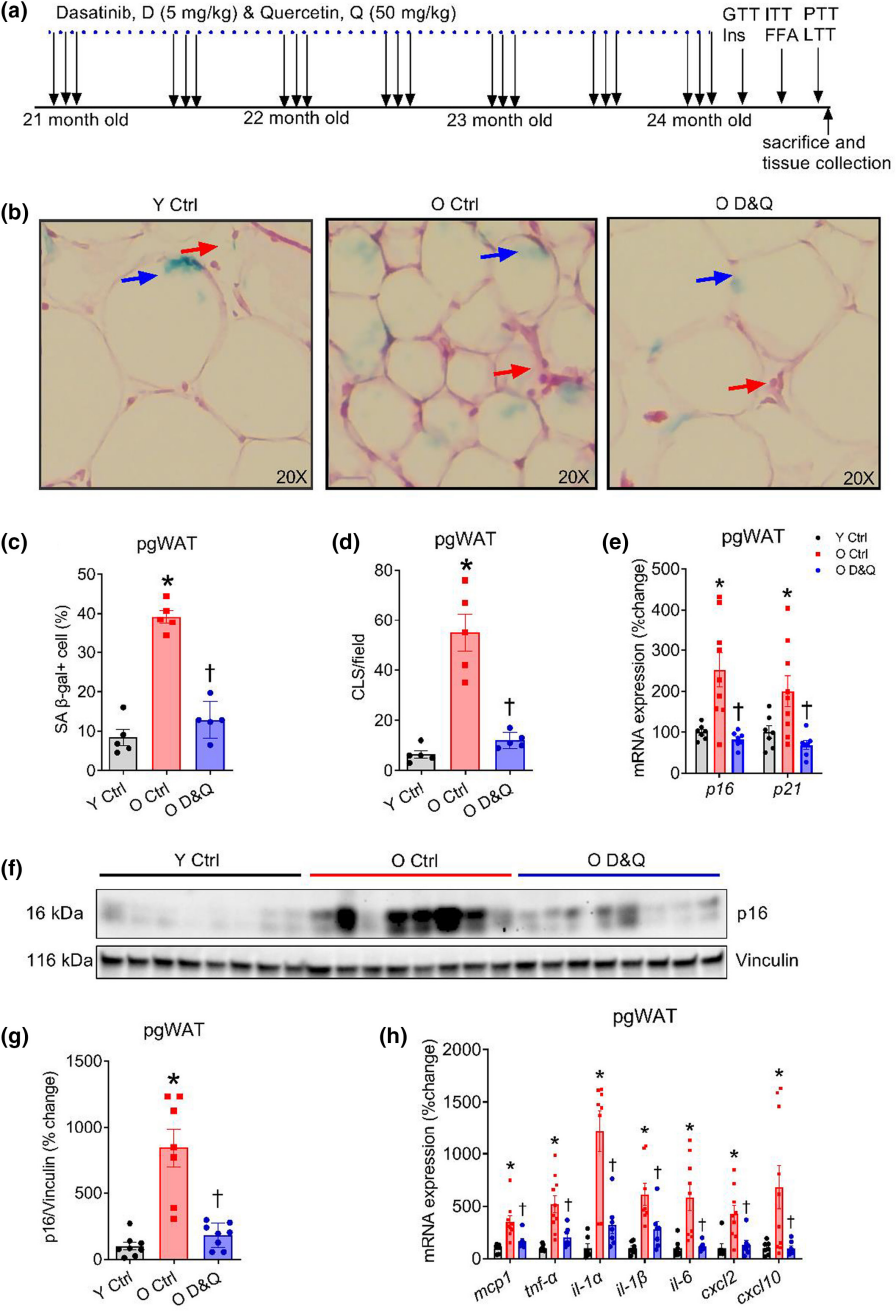
Administration of D&Q suppresses age‐related increases in senescence and inflammatory markers in pgWAT. (a) Schematic representation of the dasatinib and quercetin (D&Q) treatment and the non‐terminal experiments such as glucose‐, insulin‐, pyruvate‐, and intralipid‐tolerance tests (GTT, ITT, PTT, and LTT), insulin secretion during GTT, insulin‐stimulated free fatty acid suppression (Ins‐FFA) measurement, (b) representative H&E images of perigonadal white adipose tissue (pgWAT) after staining with senescence‐associated β‐galactosidase (SA β‐gal), blue arrowheads indicate SA β‐gal+ cells and red arrowheads indicate crown‐like structures (CLS) (c and d) quantification of SA β‐gal+ cells and CLS, (e) *p16* and *p21* gene expression relative to *18s* in pgWAT, (f and g) Western blot images and quantification of P16 protein expression relative to vinculin, (h) *mcp1, tnf‐α, il‐1α, il‐1β, il‐6, cxcl2, and cxcl10* gene expression relative to *18s* in pgWAT. Data are shown as mean ± SEM with individual data points in the bar graphs. *N* = 5–8/group. *Denote *p* ≤ 0.04 versus Y Ctrl, ^†^denote *p* ≤ 0.04 versus O Ctrl. Group differences were assessed by one‐way ANOVA with Tukey's post hoc tests.

Twenty‐one‐month‐old mice received the senolytic drugs dasatinib (5 mg/kg body mass) and quercetin (50 mg/kg body mass) on three consecutive days, every 2 weeks for 3 months via oral gavage (Figure 1a). Control mice were treated with vehicle (10% polyethylene glycol 4000 solution) only.

### 2.3 Metabolic testing

Metabolic assays were performed as described previously (Trott et al., 2021). For glucose, insulin, and pyruvate tolerance tests (GTT, ITT, and PTT), mice were fasted 5–6 h in the morning and a baseline blood glucose was measured using Precision Xceed Pro Glucometer in blood collected via tail nick. An additional 40–50 μl blood was collected for measurement of plasma insulin. Mice were injected with either dextrose (2 g/kg, i.p., GTT), insulin (1 U/kg, i.p., ITT), or sodium pyruvate (2 g/kg, i.p., PTT), and blood glucose was measured at 15, 30, 60, 90, and 120 min after injection. During the GTT, 15 min after the glucose injection, 20–30 μl blood was collected for measuring plasma insulin at baseline and during GTT. Likewise, during the ITT, 15 min after the insulin injection, ~10 μl blood was collected to measure plasma non‐esterified fatty acids (NEFAs) allowing us to examine insulin‐stimulated suppression of plasma NEFAs. To measure plasma triglycerides, 20–30 μl blood was collected in the morning between 7 am to 7:30 am. For lipid‐tolerance test (LTT), mice were fasted overnight and ~10 μl blood was collected via tail nick in the morning, Then, a bolus 20% intralipid solution (Sigma) were administered (15 μl/g body mass) via oral gavage. Approximately 10 μl blood was collected again at 30, 60, 120, and 180 min after intralipid administration. Blood for plasma separation was collected into heparinized Microvette® CB 300 (Sarstedt) via tail nick and centrifuged at 7500*g* for 5 min at 4°C. Plasma was collected and stored at −80°C for subsequent analysis.

### 2.4 Enzyme‐linked immunosorbent assay

Plasma insulin was measured using Ultra‐Sensitive Mouse Insulin ELISA Kit (Chrystal Chem) according to the manufacturer's protocol.

### 2.5 Plasma lipids measurement

Plasma non‐esterified fatty acids were measured using a commercially available enzymatic colorimetric assay kit (FUJIFILM Wako Chemicals) according to the manufacturer's protocol. Likewise, plasma triglycerides were measured using enzymatic colorimetry‐based Infinity™ Triglycerides Liquid Stable Reagents (Thermo Fisher) following the manufacturer's protocol. Total cholesterol, total triglycerides, LDL, and HDL levels were measured using dedicated on‐board reagents from Abbott Laboratories (Cat#7D62‐21, 7D74‐21, 1E31‐20, and 3k33‐22).

### 2.6 Flow cytometry

Following euthanasia, the chest cavity was opened, and the right atrium was nicked. To remove circulating T cells, a cannula was placed in the left ventricle and the animals were perfused with saline +10 U/ml of heparin at physiological pressure until the effluent was cleared of blood. Adipose tissue was excised, weighed, and digested using collagenase type I (2 mg/ml), and DNAse (0.1 mg/ml) dissolved in phosphate‐buffered saline containing calcium and magnesium for 30–60 min at 37°C. The tissues were further dispersed using repeated pipetting and the resultant homogenate was passed through a 70‐μm sterile filter, yielding single‐cell suspensions. Single‐cell suspensions were labeled with the following anti‐mouse antibodies at a 1:100 concentration: violetfluor450‐CD45, Tonbo #75‐0454 (total leukocytes); 1:100 concentration: APC‐CD3, Tonbo #20‐0032 (pan T cells). Dead cells were labeled with violetFlour510 Ghost Dye (Tonbo) and excluded from analysis. Cell subpopulations were assessed on a BD FACS Canto. The “fluorescence minus one” technique was used to establish gating, as described previously (Trott et al., 2018). Macrophages were assessed as described previously (Trott et al., 2021) with violetfluor450‐CD45 (total leukocytes); APC Cy7‐CD19, Biolegend #115529 (B cells); PE‐CD64, Biolegend #139303 (macrophages); FITC‐CD11c, Tonbo #35‐0114 (exclusion of dendritic cells); and APC‐CD206, Biolegend #141707 (macrophage phenotype).

### 2.7 Quantitative polymerase chain reactions

Assessment of gene expression was performed as described previously (Machin et al., 2020). Total mRNA was extracted from frozen liver, gastrocnemius muscle, and visceral, perigonadal adipose tissue using RNeasy Mini Kit (Qiagen) according to the manufacturer's protocol. cDNA was synthesized from 800 ng of total mRNA using QuantiTect Reverse Transcription Kit (Qiagen) according to the manufacturer's protocol. Quantitative PCR was performed on 96‐well plates using SsoFast™ EvaGreen^R^ Supermix (Bio‐Rad) with the Bio‐Rad CFX™ Real‐Time System. Expression of the genes was normalized to 18s and fold change was calculated using the ΔΔCt method. Primer sequences were as follows: *18s* F 5′‐TAGAGGGACAAGTGGCGTTC‐3′, *18s* R 5′‐CGCTGAGCCAGTCAGTGT‐3′; *p16* F 5′‐CGCAGGTTCTTGGTCACTGT‐3′, *p16* R 5′‐TGTTCACGAAAGCCAGAGCG‐3′; *p21* F 5′‐CCTGGTGATGTCCGACCTG‐3′, *p21* R 5′‐CCATGAGCGCATCGCAATC‐3′; *mcp1* F 5′‐GCATCCACGTGTTGGCTCA‐3′, *mcp1* R 5′‐CTCCAGCCTACTCATTGGGATCA‐3′; *cxcl2* F 5′‐CCTGGTTCAGAAAATCATCCA‐3′, *cxcl2* R 5′‐CTTCCGTTGAGGGACAGC‐3′; *cxcl10* F 5′‐TCATCCTGCTGGGTCTGAGT‐3′, *cxcl10* R 5′‐ATCGTGGCAATGATCTCAACAC‐3′; *il‐6* F 5′‐CTGGGAAATCGTGGAAT‐3′, *il‐6* R 5′‐CCAGTTTGGTAGCATCCATC‐3′; *il‐1α* F 5′‐CGAAGACTACAGTTCTGCCATT‐3′, *il‐1α* R 5′‐GACGTTTCAGAGGTTCTCAGAG‐3; *il‐1β* F 5′‐CACAGCAGCACATCAACAAG‐3′, *il‐1β* R 5′‐GTGCTCATGTCCTCATCCTG‐3′; *tnf‐α* F 5′ ATGAGAAGTTCCCAAATGGC‐3′, *tnf‐α* R 5′‐CTCCACTTGGTGGTTTGCTA‐3′; *cd3e* F 5′‐GACTATGAGCCCATCCGCAAA‐3′, *cd3e* R 5′‐TAGGACACGTGTTCACCAGGA‐3′; *foxp3* F 5′‐GGCCCTTCTCCAGGACAGA‐3′, *foxp3* R 5′‐GCTGATCATGGCTGGGTTGT‐3′; *cpt1α* F 5′‐CTCCGCCTGAGCCATGAAG‐3′ *cpt1α* R 5′‐CACCAGTGATGATGCCATTCT‐3′, *pck1* F 5′‐CCTGGAAGAACAAGGAGTGG‐3′, *pck1* R 5′‐AGGGTCAATAATGGGGCACT‐3′; *pck2* F 5′‐CCCTATCACAAGGCAAGAGA‐3′, *pck2* R 5′‐CCACTTCCCCTGTCCTATTT‐3′; *fbp2* F 5′‐GGGGGAAATATGTGGTTTGCT‐3′, *fbp2* R 5′‐TCCTCCGTGGTCTTTCTGTAAA‐3′; *g6pc* F 5′‐GTCTGGATTCTACCTGCTAC‐3′, *g6pc* R 5′‐AAAGACTTCTTGTGTGTCTGTC‐3′; *irs1* F 5′‐TCCCAAACAGAAGGAGGATG‐3′, *irs1* R 5′‐CATTCCGAGGAGAGCTTTTG‐3′; *fgf21* F 5′‐CTGCTGGGGGTCTACCAAG‐3′, *fgf21* R 5′‐CTGCGCCTACCACTGTTCC‐3′; *pparα* F 5′‐AGAGCCCCATCTGTCCTCTC‐3′, *pparα* R 5′‐ACTGGTAGTCTGCAAAACCAAA‐3′.

### 2.8 Western blots

Mice were fasted for 2 h before euthanasia. After euthanasia, tissues were harvested, frozen in liquid nitrogen, and stored at −80°C. Protein lysates were prepared from liver using ice‐cold RIPA buffer (Sigma Aldrich) with proteases and phosphatase inhibitor cocktails (Thermo Fisher). Protein concentration was measured using BCA assay (Thermo Fisher) according to the manufacturer's protocol. Protein expression was measured by standard western blot procedures using anti‐mouse primary antibodies against p16 (CDKN2A/p16INK4a; 1:1000; 16 kDa; Abcam #211542), total Akt (pan‐Akt; 1:1000; 60 kDa; cell signaling), phosphorylated Akt (s‐473 p‐Akt; 1:1000; 60 kDa; cell signaling), total CREB (CREB; 1:1000; 43 kDa; Cell Signaling), phosphorylated CREB (s‐133 p‐CREB; 1:1000; 43 kDa; Abcam), and vinculin (hVIN‐1, monoclonal; 1:1000; 116 kDa, Sigma Aldrich). Goat Anti‐Rabbit IgG (H + L)‐HRP Conjugate (Bio‐Rad) and Goat Anti‐Mouse IgG (H + L)‐HRP Conjugate (Bio‐Rad) were used as the secondary antibody. Images were visualized and quantified using Bio‐Rad ChemiDoc™ XRS+ with Image Lab™ Software.

### 2.9 Adipose tissue histology

Adipose tissue histology and senescence‐associated β‐galactosidase (SA β‐gal) staining were performed as described previously (Xu, Tchkonia, et al., 2015). Briefly, a small piece of perigonadal adipose tissue was collected in phosphate‐buffered saline (PBS), lightly fixed with 2% paraformaldehyde (PFA) and 0.25% glutaraldehyde for 1 h, washed three times in PBS and placed in SA β‐gal activity solution containing X‐gal at pH 6.0 at 37°C for 18–20 h. Samples were washed three times in PBS and fixed with 4% PFA overnight, typically 12–16 h, at 4°C, washed three times in PBS and transferred to 70% ethanol solution until paraffin embedding. Paraffin‐embedded samples were sliced into 8 μm sections and stained with hematoxylin and eosin (H&E) according to the manufacturer's instructions. Four to five images per samples were captured using EVOS light microscope with 20X magnification. SA β‐gal+ cells as a percent of all cells were quantified by an examiner blinded to the treatment group. Crown‐like structures were also counted as a marker of inflammation.

### 2.10 Liver histology

Following euthanasia, a lobe of the liver was dissected, washed in PBS, and fixed in 4% PFA solution for 48 h. After the fixation, samples were washed in PBS and transferred to 70% ethanol solution until paraffin embedding. Paraffin‐embedded samples were sliced into 5 μm sections and stained with hematoxylin and eosin (H&E) according to the manufacturer's instructions. H&E‐stained slides were scanned and assessed by an examiner, typically a veterinary pathologist, blinded to the treatment condition. Five to six samples were analyzed from each experimental group. To examine the collagen deposition, 5 μm sections were stained with picrosirius red (PSR) according to manufacturer's protocol. Slides were scanned and the pixel positivity for PSR were quantified using the Image J FIJI software.

### 2.11 Statistics

Statistical analyses were performed using GraphPad Prism software, and data are presented as mean ± SEM with individual data points where appropriate. Most of the differences between vehicle and D&Q‐treated mice were assessed by two‐way ANOVA or student's *t* test. Differences in glucose, insulin, and pyruvate tolerance tests were assessed by repeated measure ANOVA with Tukey's LSD post hoc tests. Statistical significance was set at p < 0.05.

## 3 RESULTS

### 3.1 Effects of advanced age and D&Q on body and tissue mass

Body mass of the old mice was higher compared with young mice and the administration of D&Q reduced the body mass in old mice (both *p* ≤ 0.05; Table 1). Likewise, perigonadal white adipose tissue (pgWAT) mass was higher in old compared with young mice and D&Q treatment reduced pgWAT mass in old mice (both *p* ≤ 0.04; Table 1). Subcutaneous white adipose tissue (scWAT) mass was lower in old compared with young mice and D&Q treatment increased scWAT mass in old mice (both *p* ≤ 0.001; Table 1). Kidney mass was higher in old compared with young mice (*p* = 0.02; Table 1) and D&Q did not alter kidney mass in old mice (*p* = 0.53; Table 1). Although age did not alter heart mass (*p* = 0.97; Table 1), D&Q increased heart mass in old mice (*p* = 0.05; Table 1). We did not find any difference in the mass of the liver, skeletal muscles, or spleen (all *p* ≥ 0.11; Table 1).

**TABLE 1:** Body and tissue mass of young control, old control, and old D&Q‐treated mice

	Y Ctrl	O Ctrl	O D&Q
Body mass (g)	29.0 ± 0.8	31.3 ± 0.6*	29.4 ± 0.7^†^
Liver mass (g)	1.53 ± 0.1	1.58 ± 0.1	1.51 ± 0.1
pgWAT (mg)	310 ± 12	403 ± 27*	315 ± 32^†^
scWAT mass (mg)	387 ± 23	244 ± 24*	421 ± 24^†^
Gastrocnemius mass (mg)	257 ± 13	256 ± 9	260 ± 9
Quadriceps mass (mg)	313 ± 19	326 ± 9	304 ± 11
Kidney mass (mg)	201 ± 11	231 ± 7*	224 ± 9
Heart mass (mg)	153 ± 7	154 ± 4	171 ± 6^†^
Spleen mass (mg)	104 ± 7	99 ± 4	101 ± 8

*Note*: Data are shown as mean ± SEM. N: Y Ctrl = 8, O Ctrl = 15, O D&Q = 17.

^†^ Denotes *p* ≤ 0.04 vs. O Ctrl.

* Denotes *p* ≤ 0.04 vs. Y Ctrl.

### 3.2 Advanced age and D&Q treatment demonstrated differential impacts on senescence and inflammatory markers in pgWAT, liver, and muscle

We first sought to examine the effects of advanced age and D&Q treatment on senescence and inflammatory senescence‐associated secretory phenotype (SASP) factor burden in metabolically active tissues such as pgWAT, liver, and gastrocnemius muscle. To do so, we performed histological assessments as well as measured gene and protein expression for senescence and a subset of inflammatory SASP markers in tissue lysates. Aging increased senescence‐associated β‐galactosidase (SA β‐gal) + cells and crown‐like structures (CLS) in pgWAT, and these were lower in pgWAT of D&Q‐treated old mice (all *p* ≤ 0.001; Figure 1b–d). Likewise, aging increased the gene expression of *p16* and *p21* in pgWAT, which was reduced by D&Q treatment (all *p* ≤ 0.04; Figure 1e). Advanced age also increased P16 protein expression and D&Q treatment reduced P16 (both *p* ≤ 0.001; Figure 1f,g). Gene expression of a subset of inflammatory SASP markers such as *mcp1, tnf‐α, il‐1α, il‐1β, il‐6, cxcl2*, and *cxcl10* was higher in pgWAT from old compared with young mice and D&Q treatment reduced these markers in pgWAT from old mice (all *p* ≤ 0.03; Figure 1h).

Gene expression for *p16* was higher in liver of old compared with young mice and D&Q treatment reduced *p16* gene expression in liver of old mice (both *p* ≤ 0.003; Figure S1a). Gene expression for *p21* in liver was not influenced by age or D&Q (both *p* ≥ 0.67; Figure S1a). Advanced age increased p16 protein expression in liver (*p* = 0.03), however, D&Q treatment did not alter P16 protein expression in the liver of old mice (*p* = 0.83; Figure S1b,c). Likewise, advanced age increased the gene expression of inflammatory SASP markers *mcp1, tnf‐α*, and *il‐1β* in the liver (all *p* ≤ 0.03; Figure S1d). D&Q reduced the expression of *mcp1* (*p* = 0.04), but not *tnf‐α* or *il‐1β* (both *p* ≥ 0.31; Figure S1d). Finally, treatment with D&Q did not alter gene expression of senescence or inflammatory markers in the gastrocnemius muscle (all *p* ≥ 0.19; Figure S1e,f). Taken together, these results suggest that advanced age increases senescence and inflammatory SASP burden in the pgWAT and liver. Treatment with D&Q reduces age‐related increases in senescence and inflammatory burden in the adipose tissue and demonstrates no major effects in the liver and skeletal muscle.

### 3.3 D&Q reduced the age‐related increase in the abundance of T lymphocyte and macrophages in pgWAT

**FIGURE 2:**
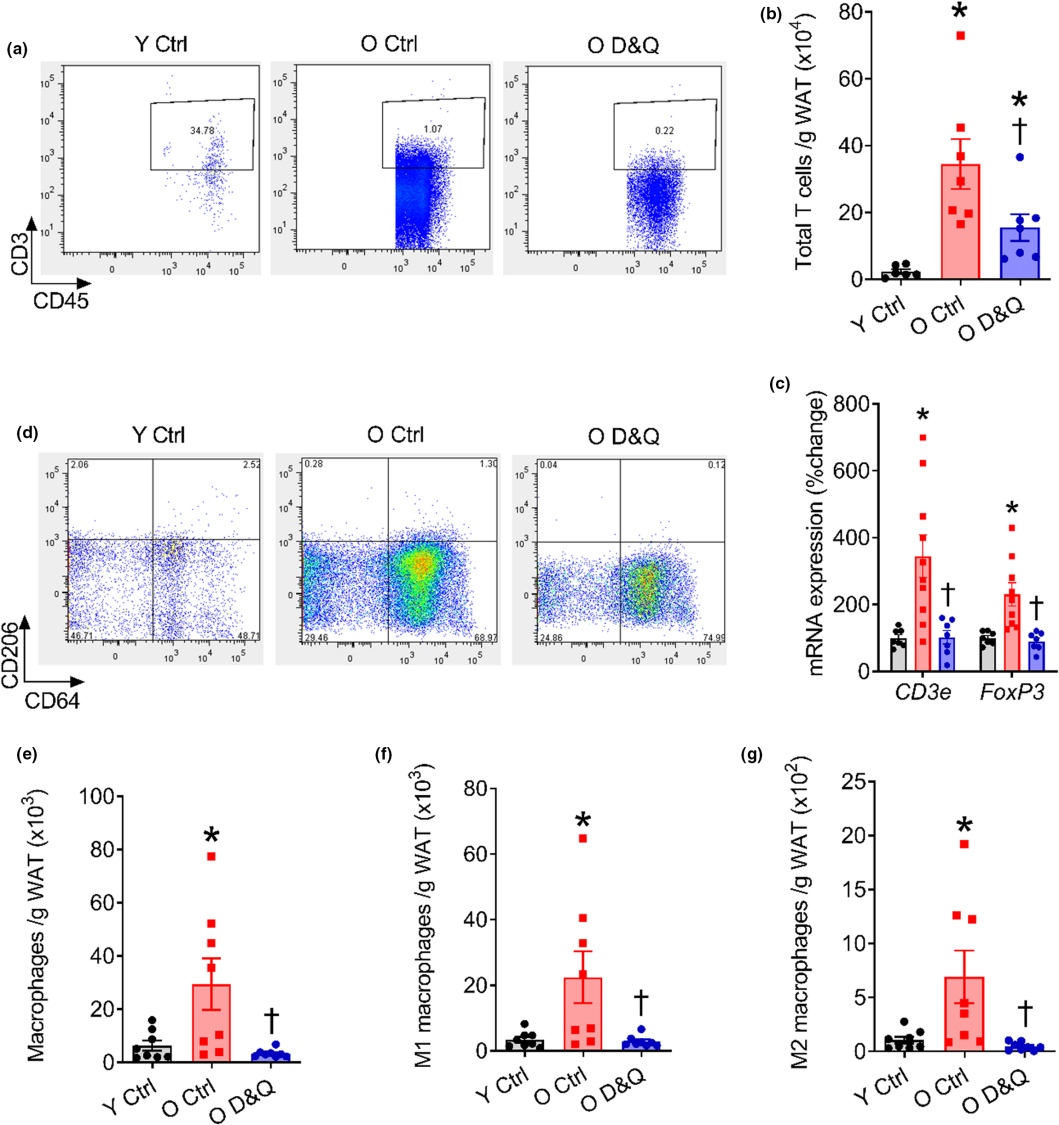
Suppressed senescence and inflammatory burden is concomitant to reduced T cells and macrophages in pgWAT. (a and b) representative images and quantification of total T cells (CD45+, CD3+) in perigonadal white adipose tissue (pgWAT), (c) *CD3e* and *FoxP3* gene expression relative to 18s in pgWAT, (d–g) representative images and quantification of total, M1 (CD206−) and M2 (CD206+) macrophages in pgWAT. Data are shown as mean ± SEM with individual data points in the bar graphs. *N* = 7/group. *Denote *p* ≤ 0.01 versus Y Ctrl, ^†^denote *p* ≤ 0.04 versus O Ctrl. Group differences were assessed by one‐way ANOVA with Tukey's post hoc tests.

To examine the relation between senescence and inflammatory SASP burden with immune cells, we assessed the abundance of T cells and macrophages in pgWAT using flow cytometry. The T cells and macrophages gating strategies are shown in Figure S2a,b. Because we found that the D&Q treatment reduced the expression of senescence and inflammatory markers predominantly in the pgWAT, but not liver and skeletal muscle, we performed T cells and macrophages counting only in the pgWAT. Advanced age increased T cells (CD3+) in the pgWAT and D&Q treatment reduced the number of T cells in the pgWAT of old mice (both *p* ≤ 0.04; Figure 2a,b). Likewise, gene expression of the transcriptional regulators of total and regulatory T cells, *CD3e* and *Foxp3*, was higher in the pgWAT from old compared with young mice and D&Q treatment reduced *CD3e* and *FoxP3* gene expression in the pgWAT of old mice (all *p* ≤ 0.02; Figure 2c). Additionally, aging increased total, pro‐inflammatory (M1), and anti‐inflammatory (M2) macrophages in the pgWAT and D&Q treatment reduced the abundance of macrophages in the pgWAT of old mice (all *p* ≤ 0.01; Figure 2d–g). Thus, our findings demonstrate that advanced age increases the numbers of T cells and macrophages in the pgWAT and treatment with D&Q reverses these age‐related increases in immune cells in the pgWAT.

### 3.4 D&Q reduced fasting blood glucose and improved glucose tolerance in old mice

**FIGURE 3:**
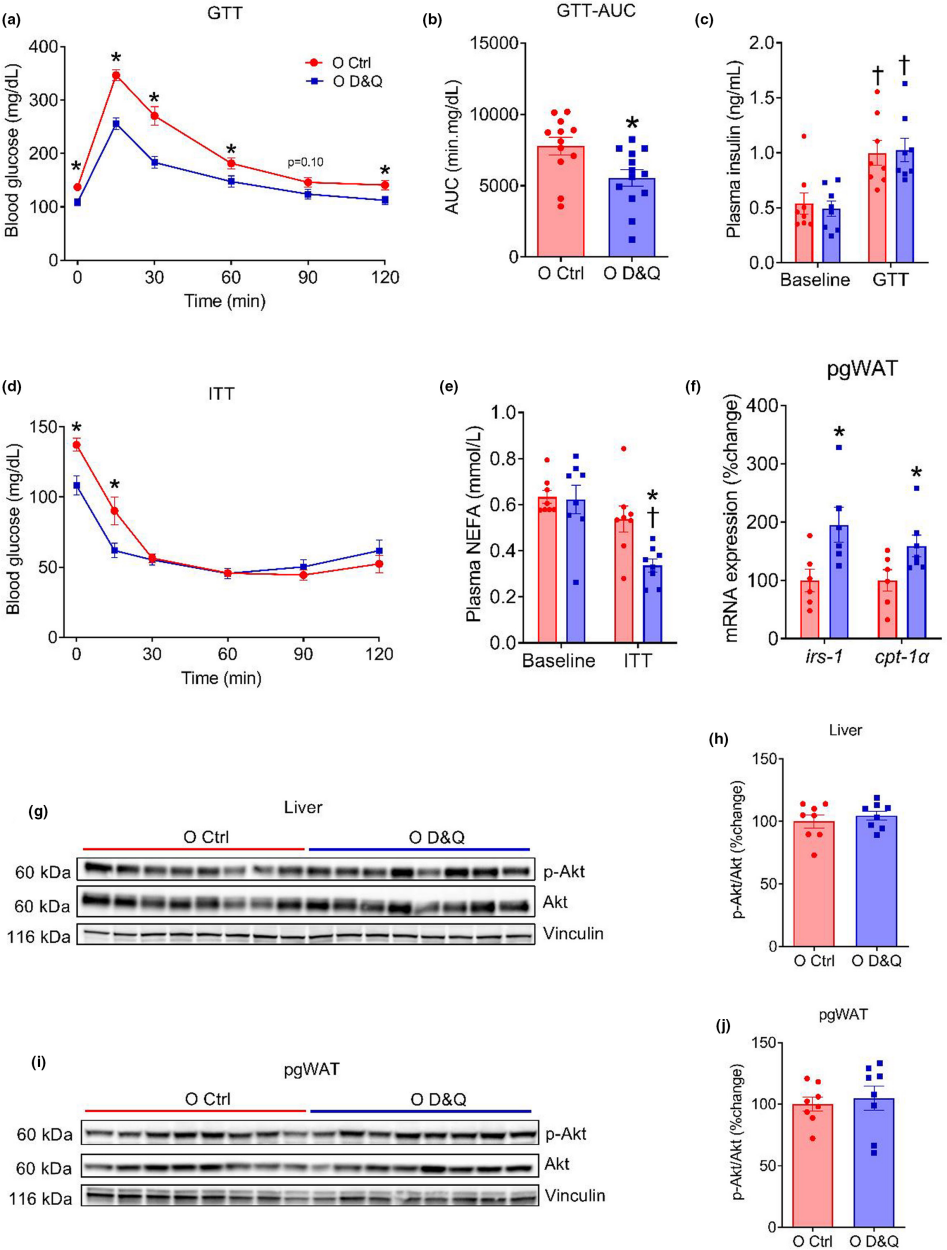
Administration of D&Q ameliorates glucose tolerance and insulin‐stimulated NEFA suppression independent of the Akt signaling. (a and b) Blood glucose response curves and area under the curves (AUC) during glucose tolerance test (GTT: 2 g/kg, ip), (c) plasma insulin at baseline and during GTT, (d) blood glucose response during insulin tolerance test (ITT: 1 U/kg, ip), (e) plasma non‐esterified fatty acids (NEFA) at baseline and during ITT, (f) *irs‐1* and *cpt‐1α* gene expression relative to *18s* in pgWAT, (g–j) Western blot images and quantification for total Akt, phosphorylated Akt and vinculin form the liver and pgWAT. Data in the time response curves are shown as mean ± SEM. Other data are shown as mean ± SEM with individual data points in the bar graphs. *N* = 7–12/group. *Denote *p* ≤ 0.03 versus O Ctrl, ^†^denote *p* ≤ 0.02 versus baseline. Group differences in GTT, ITT and plasma insulin at baseline and during GTT as well as plasma NEFA at baseline and during ITT were assessed by two‐way repeated measure ANOVA with Tukey's post hoc tests. Unpaired Student's *t* tests were used when comparing two groups.

To assess the impact of advanced age and D&Q treatment on systemic metabolic function, we first measured fasting blood glucose and performed a glucose tolerance tests (GTT) in young control, old control, and D&Q‐treated old mice. Although advanced age did not alter fasting blood glucose (*p* = 0.74) and glucose tolerance (interaction, *p* = 0.21; age, *p* = 0.18; Figure S3a), D&Q treatment reduced fasting blood glucose (*p* = 0.002) and improved glucose tolerance in old mice (interaction, *p* ≤ 0.0001; treatment, *p* ≤ 0.0001; Figure 3a). When the GTT data were expressed as percentage changes from baseline, the blood glucose at 30 min was lower in old D&Q‐treated compared with old control mice (*p* = 0.04; Figure S3b). Likewise, the baseline blood glucose‐adjusted area under the curve during the GTT was lower in old D&Q‐treated compared with old control mice (*p* = 0.0004; Figure 3b). During the GTT, we observed an expected increase in plasma insulin concentration in both old control and old D&Q‐treated mice (both *p* ≤ 0.01); however, no difference in plasma insulin at baseline or during GTT was observed between the groups (both *p* ≥ 0.69; Figure 3c).

To examine the contribution of insulin sensitivity to the improvement in glucose tolerance, we next performed an insulin tolerance test (ITT). Blood glucose was not different at baseline, 15, 30, and 60 min time points during the ITT between young and old control mice (all *p* ≥ 0.09; Figure S3c), suggesting no difference in peripheral insulin sensitivity. However, at 90 and 120 min time points, old mice demonstrated lower blood glucose compared with young control (both *p* ≤ 0.03; Figure S3c), indicating an impairment in the ability to restore blood glucose after the initial decline during ITT. When we compared old control and old D&Q‐treated mice, we found a difference in blood glucose at baseline and at 15 min during ITT (both *p* ≤ 0.02; Figure 3d), but no difference in later time points (all *p* ≥ 0.37) or overall time response curve between the groups (interaction, p˂0.0001; treatment, *p* = 0.19; Figure 3d). When the ITT was expressed as percentage change from baseline blood glucose, the difference in blood glucose at 15 min during ITT was abolished (*p* = 0.24) and at the later time points, the D&Q‐treated mice demonstrated higher blood glucose compared with old control mice (all *p* ≤ 0.05; Figure S3d), enhancing the ability to restore blood glucose after the initial decline during ITT. Taken together, our findings indicate that the senolytic drug cocktail D&Q reduces fasting blood glucose and improves glucose tolerance in old age. The improvement of glucose tolerance is independent of pancreatic beta cell function or peripheral insulin sensitivity.

### 3.5 D&Q increased insulin‐stimulated suppression of plasma NEFAs in old mice

Because plasma non‐esterified fatty acids (NEFAs) play a critical role in systemic glucose metabolism, we measured fasted and insulin‐stimulated (1 U/kg, 15 min, ip) plasma NEFAs in old control and old D&Q‐treated mice. Fasted plasma NEFAs were not different between groups (*p* = 0.87). However, stimulation with insulin reduced plasma NFFAs in old D&Q‐treated mice (*p* = 0.009), but no effect of insulin was found in old control mice (*p* = 0.11; Figure 3e). We also found a higher transcript level expression of *irs‐1* and *cpt‐1α* in the pgWAT from old D&Q‐treated mice compared with old control mice (both *p* ≤ 0.04; Figure 3f). We next measured insulin‐stimulated (1 U/kg, 30 min, ip) phosphorylation of Akt in the liver and pgWAT and did not find any difference between the groups (both *p* ≥ 0.23; Figure 3g–j). Collectively, these results suggest that the D&Q administration enhances the ability of insulin to suppress plasma NEFAs, which may have contributed to the improvement in glucose metabolism although the mechanisms require further elucidation.

### 3.6 Improved glucose tolerance was associated with reduced hepatic gluconeogenesis

**FIGURE 4:**
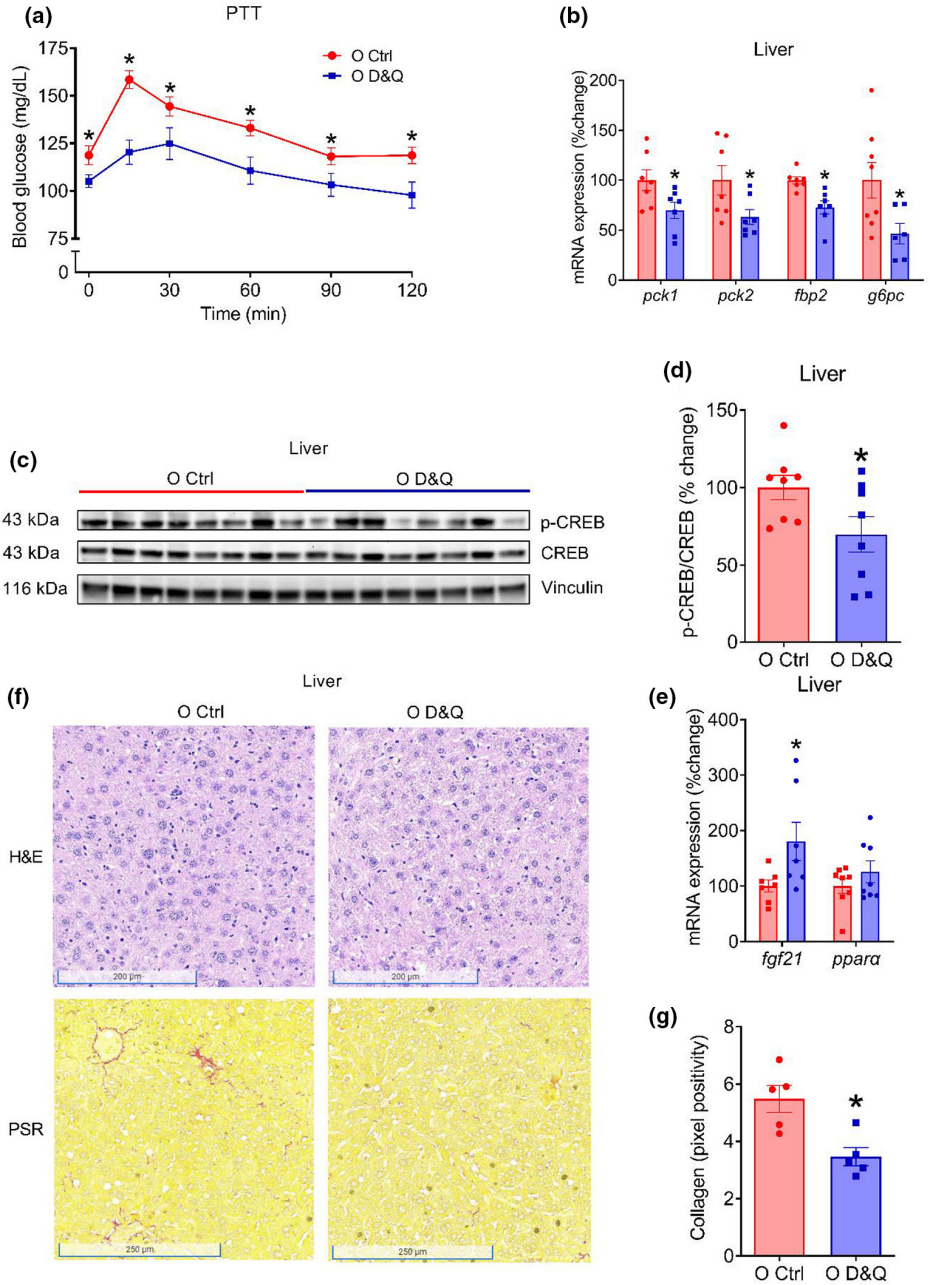
Improvement in glucose tolerance is accompanied by attenuated hepatic gluconeogenesis and collagen content. (a) Blood glucose response during pyruvate tolerance test (PTT: 2 g/kg, ip), (b) gluconeogenic gene (*pck1, pck2, fbp2, and g6pc*) expression relative to *18s* in liver, (c) representative Western blot images of phosphorylated‐ and total‐cAMP‐response element‐binding protein (CREB) as well as vinculin in liver, (d) densitometric quantification of p‐CREB and CREB, (e) *fgf21* and *pparα* gene expression relative to 18s in liver, (f) representative hematoxylin and eosin (H&E) and picrosirius red (PSR) staining of liver, (g) quantification of the pixel positivity for collagen in the liver. Data in the time response curves are shown as mean ± SEM. Other data are shown as mean ± SEM with individual data points in the bar graphs. *N* = 5–12/group. *Denote *p* ≤ 0.05. Group difference in PTT was assessed by two‐way repeated measure ANOVA with Tukey's post hoc tests. Unpaired Student's *t* tests were used when comparing two groups.

To gain insight into the contribution of hepatic gluconeogenesis to improved glucose tolerance resulted by the administration of D&Q, we performed a pyruvate tolerance test (PTT). Although, advanced age did not influence pyruvate tolerance (interaction, *p* = 0.95, age, *p* = 0.12; Figure S4a), treatment with D&Q improved blood glucose response during the PTT (interaction, *p* = 0.012, treatment, *p* = 0.004; Figure 4a), suggesting suppressed hepatic gluconeogenesis. When the PTT data were expressed as percentage changes from baseline, the blood glucose at 15 min was lower in old D&Q‐treated compared with old control mice (*p* = 0.006; Figure S4b).

To further elucidate the mechanisms that underlie reduced gluconeogenesis after D&Q administration, we examined transcript level expression of gluconeogenic genes (*pck1, pck2, fbp2*, and *g6pc*) as well as total and phosphorylated protein expression of a key transcription factor, cAMP‐response element‐binding protein (CREB). Aging increased the expression of gluconeogenic genes (all *p* ≤ 0.03; Figure S4c) and D&Q treatment reduced the expression of these genes (all *p* ≤ 04; Figure 4b). Likewise, the ratio of phosphorylated to total CREB protein was lower in D&Q‐treated mice compared with vehicle‐treated mice (*p* = 0.047; Figure 4c,d). We also examined the hepatic gene expression of important metabolic regulators, *fgf21* and *pparα*, and found that advanced age reduced the expression of *fgf21* (*p* = 0.04; Figure S4d) and D&Q treatment increases the expression of *fgf21* (*p* = 0.03; Figure 4e). Expression of *pparα* was not altered either by age or treatment (*p* ≥ 0.16; Figure S4d and Figure 4e). Taken together, these findings suggest that D&Q treatment results in lower CREB phosphorylation, leading to attenuated transcription of gluconeogenic genes and hepatic gluconeogenesis. These findings also suggest that a decline in hepatic gluconeogenesis is a major contributor to improved glucose tolerance in D&Q‐treated old mice.

### 3.7 Administration of D&Q reduced age‐related increase in collagen deposition in liver

To test the impact of aging and D&Q on the histopathological features of liver, we performed hematoxylin and eosin (H&E) and picrosirius red (PSR) staining in paraformaldehyde‐fixed and paraffin‐embedded liver sections. In the H&E‐stained slides, we did not observe any noticeable differences between D&Q‐treated and old control mice (Figure 4f). In the PSR‐stained slides, we observed an age‐related increase in collagen deposition (*p* = 0.008; Figure S3f,g), that was reduced by D&Q treatment in the old mice (*p* = 0.007; Figure 4f,g). These data suggest that the D&Q treatment reduces age‐related increases in collagen deposition in the liver that may contribute to the improvement in metabolic function such as suppressed gluconeogenesis.

### 3.8 D&Q lowered fed and fasting plasma triglycerides and improved lipid tolerance but did not alter plasma cholesterols

**FIGURE 5:**
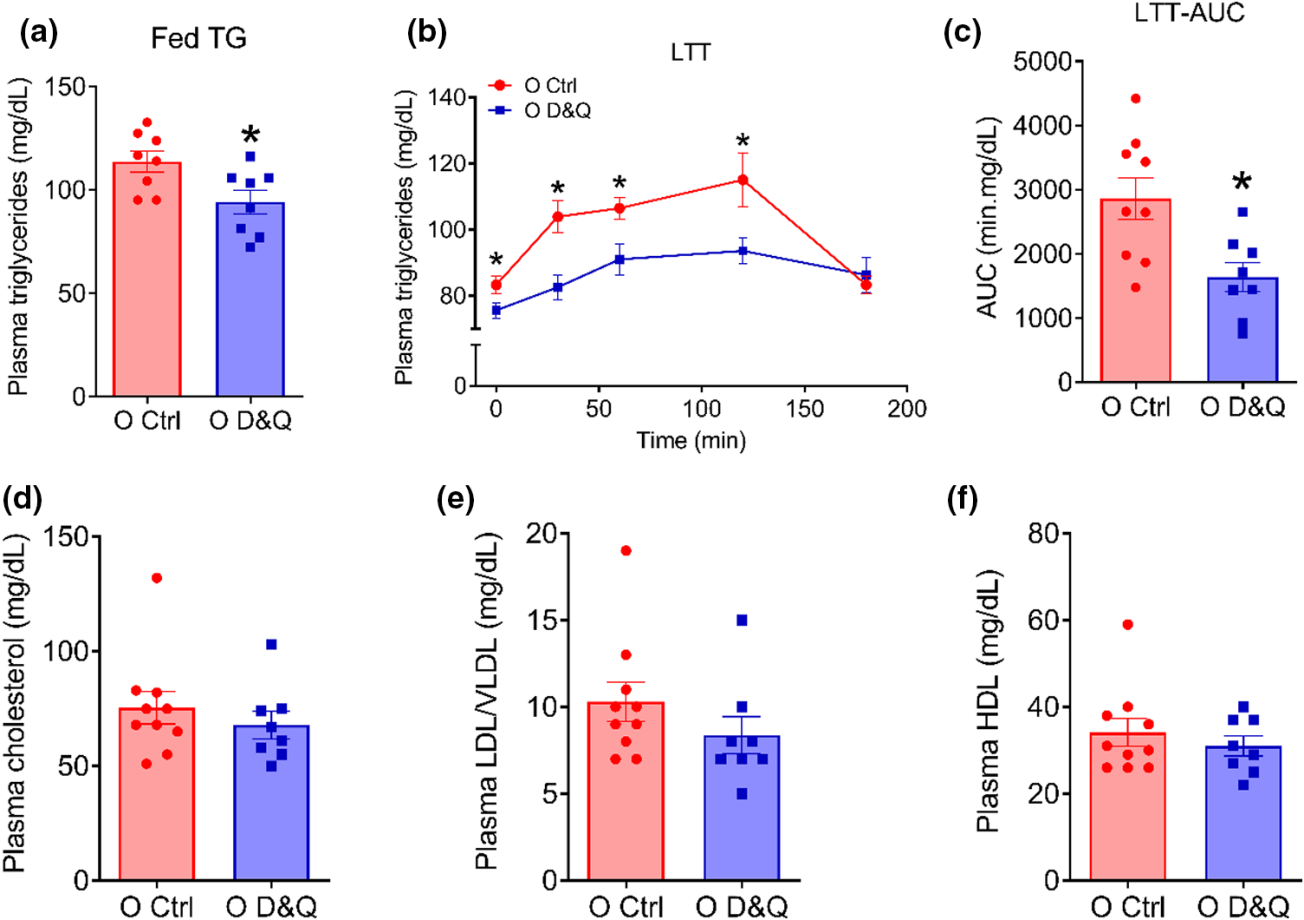
Administration of D&Q ameliorates plasma triglycerides and lipid tolerance but does not alter plasma cholesterols. (a) Fed plasma triglycerides, (b and c) plasma triglycerides response curves and area under the curves (AUC) during an intralipid‐tolerance test (LTT: 15 μl/g, oral gavage), (d) plasma cholesterol, (e) plasma low‐density and very low‐density lipoprotein, (f) plasma high‐density lipoprotein. Data in the time response curves are shown as mean ± SEM. Other data are shown as mean ± SEM with individual data points in the bar graphs. *N* = 8–10/group. *Denote *p* ≤ 0.01. Group difference in LTT was assessed by two repeated measure ANOVA with Tukey's post hoc tests. Other differences were assessed by unpaired Student's *t* test.

To examine the impact of D&Q on systemic lipid metabolism, we first measured fed and fasted plasma triglycerides. Despite no difference between young and old control mice (*p* = 12; Figure S4g), administration of D&Q reduced fed and fasted plasma triglycerides in old mice (both *p* ≤ 0.04; Figure 5a,b). We next performed a lipid‐tolerance test (LTT) and found that aging did not alter lipid tolerance (interaction, *p* = 0.46, age, *p* = 0.36; Figure S4h); however, D&Q improved systemic lipid tolerance in old mice (interaction, *p* = 0.014, treatment, *p* = 0.006; Figure 5b). When the LTT data were expressed as percent change from baseline, plasma triglyceride at 30 min was lower in old D&Q‐treated compared with old control mice (*p* = 0.01; Figure S4i). Likewise, the baseline plasma triglyceride‐corrected area under the curve (AUC) during LTT was lower in D&Q‐treated mice compared with old control mice (*p* = 0.006; Figure 5c). We also examined the impact of aging and D&Q on plasma total cholesterol, LDL/VLDL, and HDL. Advanced age did not alter total cholesterol (*p* = 0.16), but reduced HDL and increased LDL/VLDL (both *p* ≤ 0.004; Figure S4g). Treatment with D&Q did not alter plasma cholesterol, LDL/VLDL, and HDL (*p* ≥ 0.62; Figure 5d–f). Collectively, our results suggest that reducing the burden of senescence, inflammatory SASP, and adipose tissue immune cells using D&Q improves systemic lipid metabolism in old mice.

## 4 DISCUSSION

The purpose of this study was to examine the impact of the senolytic drug cocktail, dasatinib, and quercetin (D&Q) on adipose tissue inflammation and metabolic function in old age. Administration of D&Q attenuated age‐related increase in cellular senescence in perigonadal white adipose tissue (pgWAT) of old mice. This treatment also reduced the gene expression of a subset of inflammatory SASP factors in pgWAT. Attenuated burden of senescence and inflammatory SASP was concomitant with lower abundance of T cells and macrophages in the pgWAT. D&Q administration reduced fasting glucose and improved glucose tolerance in old mice. Plasma insulin during the glucose tolerance, blood glucose response during an insulin tolerance test, and insulin‐stimulated Akt phosphorylation did not differ between D&Q‐treated and control mice. However, blood glucose response during a pyruvate tolerance test, phosphorylation of c‐AMP‐response element‐binding protein, and gluconeogenic gene expression was lower in D&Q‐treated compared with control mice. Together, these results indicate that the improved glucose tolerance was primarily due to suppressed hepatic gluconeogenesis but was independent of improvements in pancreatic beta cell function or peripheral insulin sensitivity. Despite no difference in systemic insulin sensitivity, insulin‐stimulated suppression of plasma NEFAs was better in D&Q‐treated compared with control mice. Finally, D&Q administration reduced plasma triglycerides and improved lipid tolerance but did not alter plasma cholesterols. To the best of our knowledge, this is the first study demonstrating that D&Q ameliorates metabolic function in old age in a preclinical animal model. Although D&Q are currently being investigated in many different clinical trials and one trial has already been completed (clinicaltrials.gov), metabolic effects are largely absent in the outcomes of those clinical trials (Kirkland & Tchkonia, 2020). Our findings support the investigation of D&Q to treat age‐related metabolic dysfunction in humans and have the potential for rapid translation.

Advancing age results in elevated senescence and SASP burden across multiple organs and tissues (Yousefzadeh et al., 2020). Here, we demonstrate that intermittent administration of D&Q attenuates age‐related increase in the burden of senescence and a subset of inflammatory SASP in pgWAT. This treatment does not demonstrate any major effects on liver and skeletal muscle. Dasatinib is a tyrosine kinase inhibitor that primarily targets senescent adipocyte progenitor cells, while quercetin inhibits anti‐apoptotic BCL‐xL, HIF‐1α, and other senescence‐associated anti‐apoptotic proteins and eliminates senescent endothelial cells by inducing apoptosis (Kirkland & Tchkonia, 2020; Lindauer & Hochhaus, 2018; Marunaka et al., 2017; McCafferty et al., 2018; Özgür Yurttaş & Eşkazan, 2018; Vinayak & Maurya, 2019). When administered as a drug cocktail, D&Q has been demonstrated to be effective at inducing apoptosis and thus eliminating senescent cells and SASP burden in adipose tissue from both humans and mice (Hickson et al., 2019; Palmer et al., 2019; Wissler Gerdes et al., 2020). For example, intermittent administration of D&Q attenuated adipose tissue senescence burden and plasma SASP factors in human subjects with diabetic kidney disease (Hickson et al., 2019). Likewise, D&Q has been shown to reduce SA‐β‐gal+ adipocytes and *p16* gene expression in the adipose tissue from naturally aged and high‐fat diet‐fed obese mice (Palmer et al., 2019; Sierra‐Ramirez et al., 2020; Xu et al., 2018; Zhu et al., 2015). Compared with adipose tissue, the effects of D&Q on liver and skeletal muscle senescence have received less attention. However, a recent study showed that D&Q did not alter p16 protein expression in soleus muscle in a mouse model of accelerated aging which is consistent with our fundings (Ota & Kodama, 2022). Taken together, our results are in agreement with previous reports and further demonstrate novel evidence that D&Q attenuates senescence and SASP burden in a tissue‐specific manner in old age.

In addition to senescence and SASP burden, aging also results in the infiltration and accumulation of T cells in adipose tissue (Trott et al., 2021). In this study, we provide evidence that reduction in senescence and inflammatory SASP burden concomitantly attenuates the age‐related increases in the abundance of T cells and macrophages in the pgWAT of old mice. Previous studies have been demonstrated that senescent cells can recruit immune cells, amplifying inflammation. For example, Shirakawa et al. (2016) report that high‐fat diet‐induced obesity, which is known to induce adipose tissue senescence, promotes adipose tissue accumulation of T cells and macrophages. Induction of senescence in cutaneous fibroblasts in older adults resulted in infiltration of monocytes via SASP chemokine CCL2, demonstrating a direct role of senescent cells in recruiting immune cells (Chambers et al., 2021). Also, evidence exists that elimination of senescent cells can reduce adipose tissue immune cell populations (Hickson et al., 2019). In humans, reduction in adipose tissue senescence burden using D&Q has been demonstrated to reduce macrophage and crown‐like structures (Hickson et al., 2019). Likewise, in mouse model of diet‐induced obesity, clearing senescent cells using D&Q reduced adipose tissue macrophages (Palmer et al., 2019). While these studies were performed in the setting of obesity and primarily focused on macrophages, our study elucidates the impact of attenuating senescence and inflammatory SASP markers on age‐related T cells and macrophages burden in the adipose tissue.

Elevated burden of cellular senescence and SASP as well as T‐cell mediated chronic sterile inflammation contributes to a host of age‐related pathologies (Chung et al., 2019; Wissler Gerdes et al., 2020). Therefore, senescence and inflammation have emerged as potential targets for therapeutics aimed at treating a multitude of age‐related diseases, for example, diabetes, cardiovascular diseases, cancers, neurological disorders, and fibrotic diseases (Farr et al., 2017; Kirkland & Tchkonia, 2020; Palmer et al., 2019). Despite no difference in fasting blood glucose, glucose‐, pyruvate‐, and lipid tolerance between young and old mice, we demonstrate that the administration of D&Q improved metabolic function in old mice. The lack of difference in metabolic function between young and old mice is well‐evident in the literature (Biljes et al., 2015; Marmentini et al., 2021; Oh et al., 2016; Petr et al., 2021; Reynolds et al., 2019). However, a preclinical model that recapitulates every aspect of metabolic dysfunction that is observed in older human is currently unavailable. As such, the C57BL/6 mouse remains one of the most common models to study metabolic function in old age (Green et al., 2022; Martin‐Montalvo et al., 2013; Xu, Palmer, et al., 2015). It should also be noted that human aging is often compounded by environmental factors (e.g., diet, lifestyle, and microbiota) that significantly contribute to metabolic dysfunction; whereas, such confounding factors are absent in experimental mice that remain on standard rodent chow and sedentary cage activity throughout their lifespan. While further research is needed to determine the impact of aging per se on metabolic function in humans, the development and characterization of better preclinical models compared with existing models may play an instrumental role in future investigations of metabolic function in old age.

In previous studies, administration of D&Q resulted in an improvement in glucose tolerance in high‐fat diet‐fed obese mice (Palmer et al., 2019; Sierra‐Ramirez et al., 2020). However, our study is the first to demonstrate a beneficial effect of D&Q on metabolic dysfunction in old mice. Moreover, our findings suggest that the underlying mechanisms of improved glucose tolerance in old mice is distinct from what was found in obese mice. For example, studies that demonstrate D&Q improved obesity‐associated glucose tolerance did not report any findings on hepatic gluconeogenesis and one study indicated that insulin sensitivity was improved (Palmer et al., 2019; Sierra‐Ramirez et al., 2020). By contrast, our study demonstrates that the improvement in glucose tolerance was accompanied by suppressed hepatic gluconeogenesis and was independent of increased systemic insulin sensitivity or pancreatic beta cell function. Beneficial effects of reducing senescent cells and inhibiting their secreted products on metabolic function in old age has been suggested by the literature. Particularly, genetic deletion of p16 positive cells prevented age‐related loss of adipose tissue by enhancing adipogenesis in old mice (Xu, Palmer, et al., 2015). Likewise, inhibition of senescent adipocyte‐secreted product activin A using ruxolitinib improved glucose tolerance and insulin sensitivity in old mice (Xu, Palmer, et al., 2015). Our findings support these previous results and provide a more comprehensive picture of the mechanisms that underlie these improvements in metabolic function in old age after treatment with D&Q.

In addition to the improvement in glucose metabolism, we demonstrate that D&Q lowered fasted and fed plasma triglycerides and improve lipid tolerance but does not alter plasma cholesterols. Evidence demonstrating the impact of reducing senescence, SASP and immune cells on lipid metabolism is scarce in the literature. One study examined the effects of pharmacological inhibition of activin A and found that this inhibition reduced plasma‐free fatty acids, but not triglycerides, in contrast to the effects of D&Q that we observed (Xu, Palmer, et al., 2015). However, it was found that inhibition of activin A reduced hepatic triglyceride content and improved the gene expression of adipogenesis, triglyceride synthesis, and lipolysis (Xu, Palmer, et al., 2015). The impact of D&Q on plasma cholesterol in old age has not been reported previously. However, in a murine model of diet‐induced obesity, administration of D&Q did not alter plasma total cholesterol, LDL, and HDL, which is in agreement with our findings (Raffaele et al., 2021). It should be noted that cholesterol metabolism in mice is distinct from humans. For example, while human plasma contains more LDL (considered detrimental) compared with HDL (considered beneficial), mice carry more HDL compared with LDL (Emini Veseli et al., 2017). In addition, without genetic manipulation, mice do not demonstrate a robust change in plasma cholesterol with aging (Emini Veseli et al., 2017). Thus, the relevance of changes, or the lack thereof, in cholesterol in mice after D&Q to clinical populations requires further study. Taken together, our study uncovers important insight into the effects of lowering senescence, SASP, and inflammation on systemic lipid metabolism.

## 5 CONCLUSION AND FUTURE DIRECTIONS

In summary, our study demonstrates that reduction in senescence and inflammatory SASP burden attenuates the abundance of immune cells in the pgWAT and improves metabolic function in old age. Our previous work has revealed that aging results in increased infiltration of T cells in pgWAT and pharmacological depletion of T cells can improve metabolic function in old age (Trott et al., 2021). The mechanisms that underlie the increase in tissue T‐cell accumulation with aging remain poorly understood. However, it has been hypothesized that this could be due to increased SASP‐related chemokine production or immune cell dysfunction that may diminish their ability to clear senescent cells (Prata et al., 2018). Shirakawa et al. (2016), provides evidence that obesity results in the accumulation of adipose tissue senescent T cells. Therefore, it is plausible to think that D&Q may attenuate pgWAT‐resident senescent immune cells contributing to an overall reduction in the abundance of immune cells; a possibility that requires further investigation. Likewise, determining the relative contribution of senescence in individual tissue/cell types such as pgWAT and immune cells requires further investigations. Also, whether adipose tissue senescence drives immune cell infiltration and their accumulation independent of immune cell senescence or whether the induction of immune cell senescence allows for the accumulation of overall senescence burden across semi‐solid organs as seen with advancing age is of interest for future research. Transgenic mouse models of tissue‐specific gene manipulation can be instrumental in these endeavors.

## AUTHOR CONTRIBUTIONS

Md Torikul Islam and Lisa A. Lesniewski were involved in all aspects of the study including experimental design, data collection, analysis and interpretation, and manuscript preparation. Eric Tuday was involved with experimental design, data analysis, and manuscript preparation. Shanena Allen, John Kim, Daniel W. Trott, William L. Holland, and Anthony J. Donato participated in data collection and/or manuscript preparation.

## CONFLICT OF INTEREST

The authors declare no competing interests.

## Supporting information


Figure S1

## Data Availability

The data that support the findings of this study are available from the corresponding author upon reasonable request.
