# Supplementary material for: Senolytic drugs, dasatinib and quercetin, attenuate adipose tissue inflammation, and ameliorate metabolic function in old age
Source: Aging Cell. 2023 Jan 13;22(2):e13767. doi: 10.1111/acel.13767 (PMC9924942; doi:10.1111/acel.13767)
Supplement: Supplementary file 1 — Figure S1 [file ACEL-22-e13767-s001.docx]

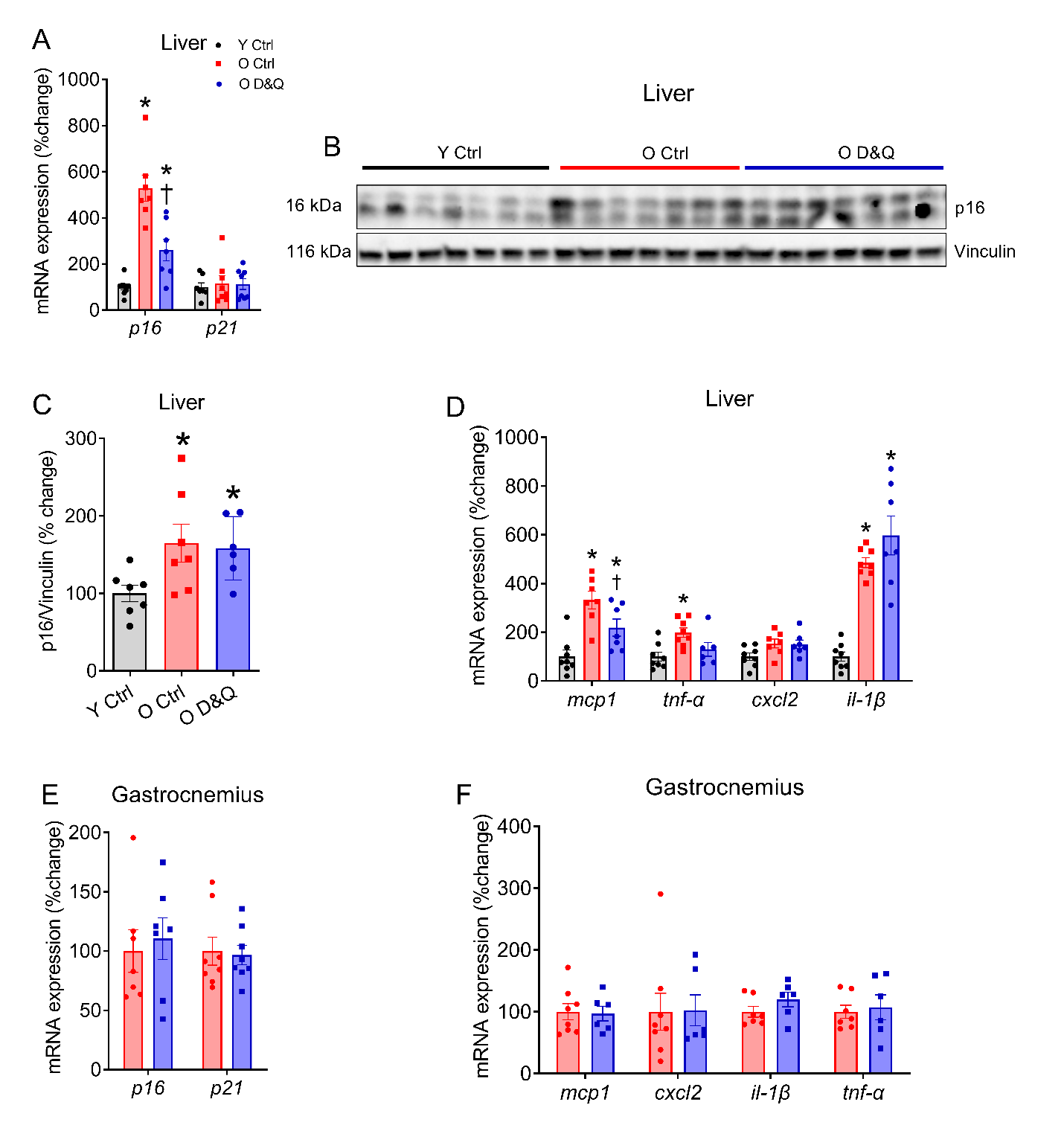


**Supplementary figure 1: Effects of D&Q on senescence and inflammatory markers in liver and gastrocnemius muscle.** (A) *p16 and p21* gene expression relative to *18s* in liver, (B, C) western blot images and quantification of p16 protein expression relative to vinculin in liver, (D) *mcp1, tnf-α, cxcl2,* and *il-1β* gene expression relative to *18s* in liver (E, F) *p16, p21, mcp1, tnf-α, cxcl2,* and *il-1β* gene expression relative to *18s* in gastrocnemius muscle. Data are shown as mean ± SEM with individual data points in the bar graphs. N=7-8/group. *Denote p≤0.05 versus Y Ctrl, †denote p ≤0.05 versus O Ctrl. Group differences were assessed by one-way ANOVA with Tukey’s post hoc tests or unpaired student's *t* tests when comparing two groups.


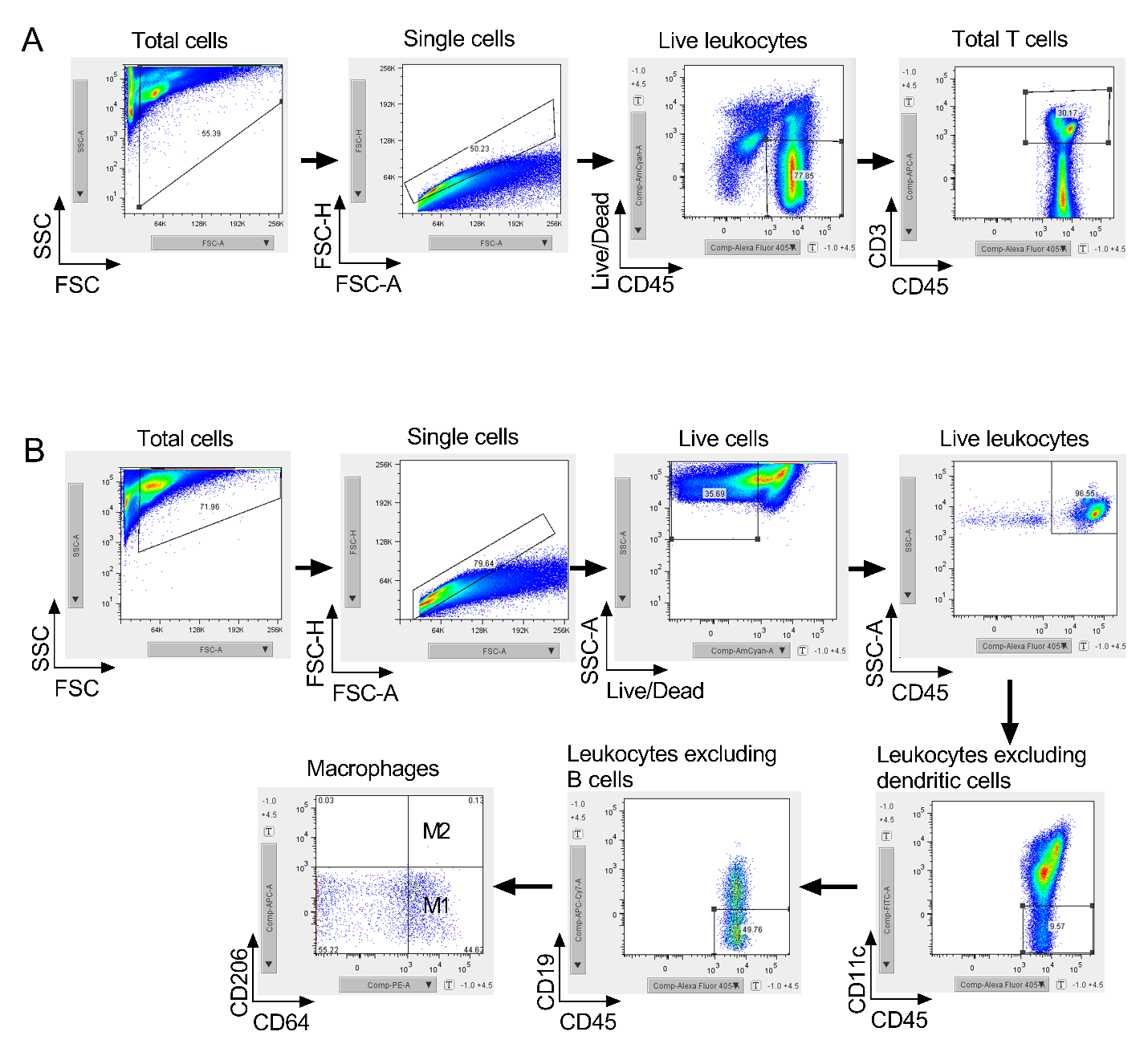


**Supplementary figure 2: Gating strategy for T cells and macrophages.** (A) After mechanical disruption and enzymatic digestion, samples were stained with anti-CD45 (leukocytes), anti-CD3 (pan T cells), and Ghost Dye (exclusion of dead cells), (B) samples were stained with anti-CD64 (macrophages), anti-CD11c (exclusion of dendritic cells) anti-CD206 (M1/M2 macrophage phenotype), anti-CD19 (B cells) and violetFluor510 Ghost Dye (exclusion of dead cells).


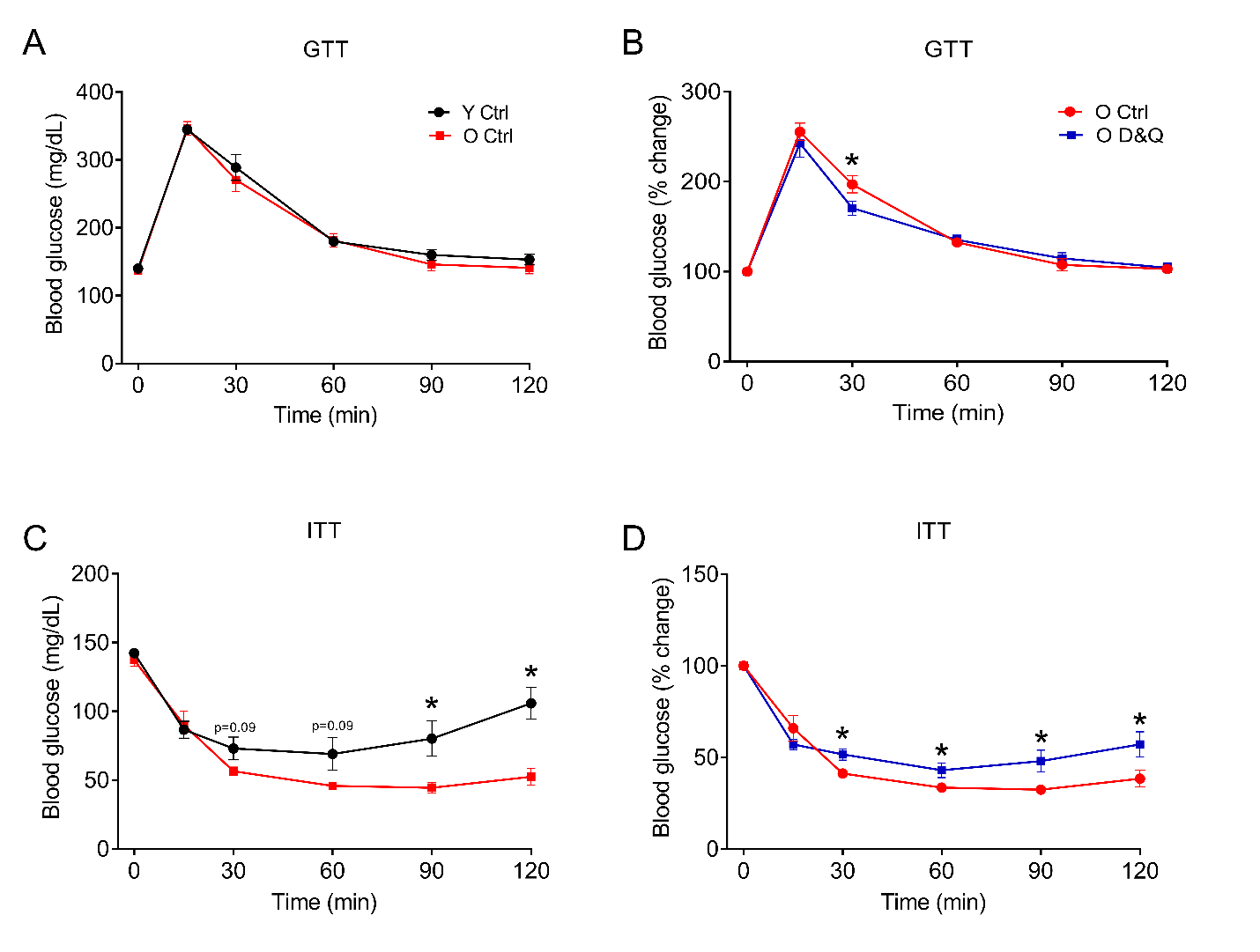


**Supplementary figure 3: Effects of advanced age and D&Q treatment on metabolic function.** (A) Blood glucose response curves during glucose tolerance test (GTT:2g/kg, ip) of young and old mice, (B) percent change of blood glucose during a GTT of old control and old D&Q treated mice, (C) blood glucose response curves during insulin tolerance test (ITT: 1U/kg, ip) of young and old mice, (D) percent change of blood glucose during ITT of old control and old D&Q treated mice. Data are shown as mean ± SEM. N=7-12/group. *Denote p≤0.05 versus Y Ctrl. Group differences were assessed by two-way repeated measure ANOVA with Tukey's post hoc tests.


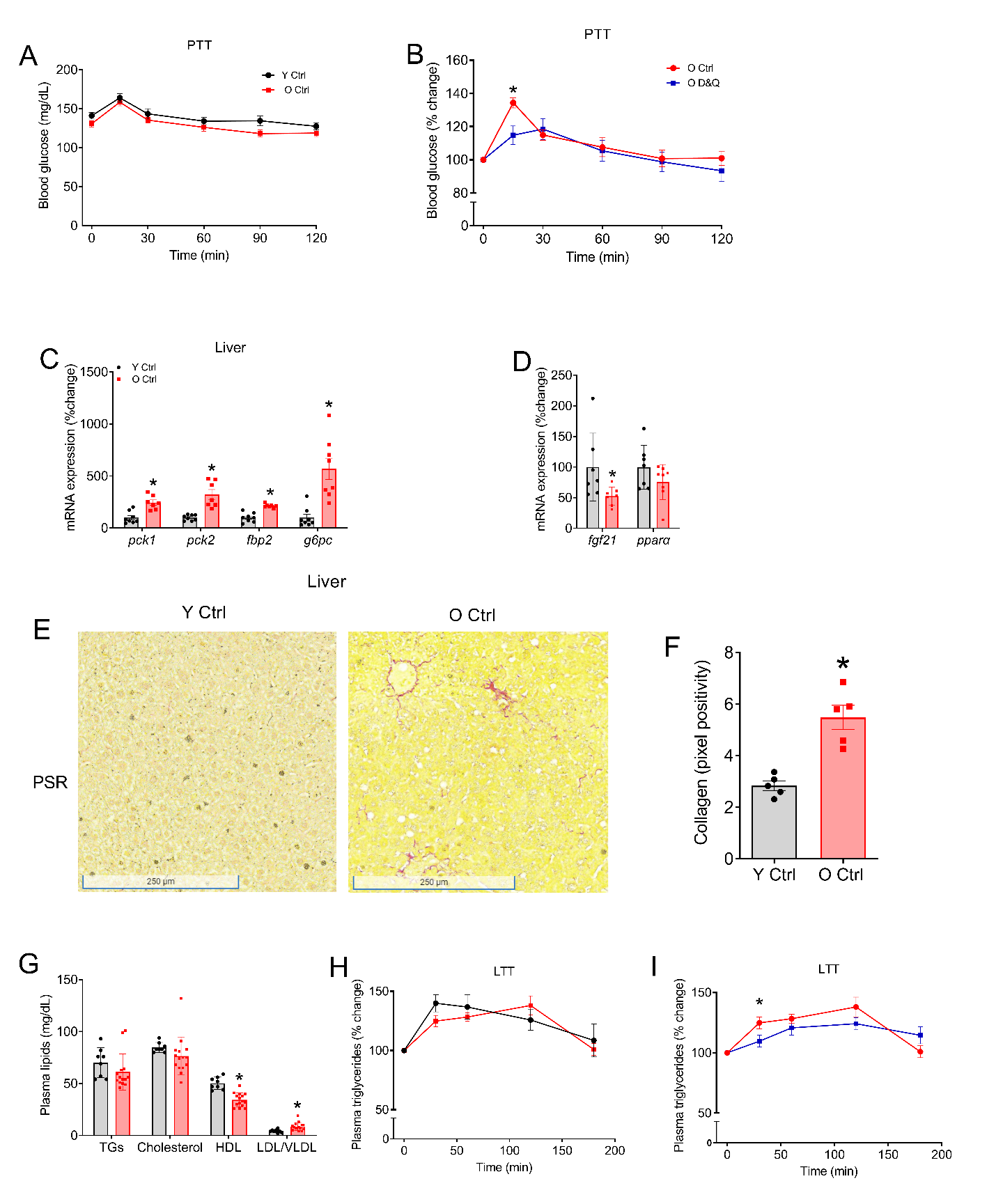


**Supplementary figure 4: Impact of advanced age and D&Q on gluconeogenesis and lipid metabolism.** (A) Blood glucose response curves during a pyruvate tolerance test (PTT:2g/kg, ip) of young and old mice, (B) percent change of blood glucose during PTT of old control and old D&Q treated mice, (C, D) *pck1, pck2, fbp2,* *g6pc, fgf21,* and *pparα* gene expression relative to *18s* from the liver of young and old mice, (E) representative picrosirius red (PSR) staining of liver, (F) quantification of the pixel positivity for collagen in the liver, (G) plasma triglycerides, total cholesterols, HDL and LDL/VLDL, (H) percent change of plasma triglycerides during an intralipid tolerance test (LTT:15μL/g, oral gavage) of young and old control mice, (I) percent change of plasma triglycerides during LTT of old control and old D&Q treated mice. Data in the curves are shown as mean ± SEM. Data in the bar graphs are shown as mean ± SEM. N=7-12/group. *Denote p≤0.04 versus Ctrl. Group differences were assessed by two-way repeated measure ANOVA with Tukey's post hoc tests or unpaired student’s *t* tests.
